# How are Long-Covid, Post-Sepsis-Syndrome and Post-Intensive-Care-Syndrome related? A conceptional approach based on the current research literature

**DOI:** 10.1186/s13054-024-05076-x

**Published:** 2024-08-29

**Authors:** Carolin Fleischmann-Struzek, Franka E. A. Joost, Mathias W. Pletz, Björn Weiß, Nicolas Paul, E. Wesley Ely, Konrad Reinhart, Norman Rose

**Affiliations:** 1 Institute of Infectious Diseases and Infection Control, Jena University Hospital, Stoystraße 3, 07743 Jena, Germany; 2 Center for Sepsis Control and Care, Jena University Hospital, Jena, Germany; 3 Center for Intervention and Research on Adaptive and Maladaptive Brain Circuits Underlying Mental Health, Jena University Hospital, Jena, Germany; 4 Department of Anesthesiology and Intensive Care Medicine (CCM, CVK), Charité – Universitätsmedizin Berlin, Corporate Member of Freie Universität Berlin and Humboldt-Universität Zu Berlin, Berlin, Germany; 5 Veteran’s Affairs Tennessee Valley Geriatric Research, Education and Clinical Center (GRECC), Nashville, TN USA; 6 Critical Illness, Brain Dysfunction, Survivorship (CIBS) Center, Vanderbilt University Medical Center, Nashville, TN USA

**Keywords:** Post-Sepsis-Syndrome, Post-covid-syndrome, Long-Covid, PICS, Post-Intensive-Care-Syndrome, IACC

## Abstract

Long-Covid (LC), Post-Sepsis-Syndrome (PSS) and Post-Intensive-Care-Syndrome (PICS) show remarkable overlaps in their clinical presentation. Nevertheless, it is unclear if they are distinct syndromes, which may co-occur in the same patient, or if they are three different labels to describe similar symptoms, assigned on the basis on patient history and professional perspective of the treating physician. Therefore, we reviewed the current literature on the relation between LC, PSS and PICS. To date, the three syndromes cannot reliably be distinguished due similarities in clinical presentation as they share the cognitive, psychological and physical impairments with only different probabilities of occurrence and a heterogeneity in individual expression. The diagnosis is furthermore hindered by a lack of specific diagnostic tools. It can be concluded that survivors after COVID-19 sepsis likely have more frequent and more severe consequences than patients with milder COVID-19 courses, and that are some COVID-19-specific sequelae, e.g. an increased risk for venous thromboembolism in the 30 days after the acute disease, which occur less often after sepsis of other causes. Patients may profit from leveraging synergies from PICS, PSS and LC treatment as well as from experiences gained from infection-associated chronic conditions in general. Disentangling molecular pathomechanisms may enable future targeted therapies that go beyond symptomatic treatment.

## Background

If patients after intensive care unit (ICU)-treated sepsis caused by COVID-19 present with new persistent sequelae > 3 months after illness, the question arises whether (i) these sequelae can be assigned to the spectrum of Long-Covid, the Post-Sepsis- or the Post-Intensive-Care-Syndrome, (ii) patients may have more than one of these syndromes or (iii) they are the same disease entity [1] and therefore cannot be assigned to a specific syndrome. In the following, overlaps and distinctions between the syndromes will be discussed based on the current research literature. For this purpose, a literature search was conducted in MEDLINE via Pubmed and Google Scholar.

### Link between Covid-19, sepsis and ICU treatment

Sepsis is a dysregulated host response to an infection that leads to organ failure [2]. Severe acute respiratory syndrome coronavirus type 2 (SARS-CoV-2) can cause viral sepsis [3], although certain differences exist with regard to the pathomechanisms underlying possible respiratory dysfunction in both diseases [4]. According to a meta-analysis from 2021, sepsis was present in one in three patients hospitalized with COVID-19 in the first phase of the pandemic, and in almost 80% of COVID-19 patients in the ICU [5]. A survey from the USA from 2020 and 2021 also found similar proportions of sepsis among hospitalized COVID-19 patients (32.5%), of which 70.8% were caused by COVID-19 alone, 26.2% by both SARS-CoV-2 and non-SARS-CoV-2 infections, and 3.1% by a bacterial infection alone [6].

Sepsis, as well as COVID-19, can be treated in both normal wards and ICUs. Even though sepsis is associated with organ dysfunction by definition and is therefore a potentially life-threatening condition, only slightly more than half of patients with sepsis are treated in the ICU in Germany and the USA [7, 8].

### Definition of Post-Intensive-Care, Post-Sepsis and Long-Covid in the literature

Sequelae after COVID-19 are referred to as Long-Covid (LC), Post-Covid-Syndrome (PCS), or Post-acute sequelae after COVID-19 (PASC). As part of an international Delphi process, the PCS was defined as the presence of symptoms in patients with probable or confirmed SARS-CoV-2 infection occurring > 3 months after the acute illness that persist for at least two months and cannot be explained by other diagnoses [9]. LC or PASC refer to all symptoms lasting for more than three months after the first symptom onset [10–12], although definitions vary. These symptoms may reappear after initial recovery from an acute COVID-19 episode, progress or persist after the initial illness [9, 12]. In the following, we will use the term LC in accordance to the recommendations of the US National Academies of Science, Engineering and Medicine [12].

Post-Sepsis-Syndrome (PSS) refers to the physical, cognitive and psychological consequences of surviving sepsis [13]. It encompasses a broad spectrum of disorders and occurs in both patients with and without intensive care treatment [14], but is more common after critical illness [14].

According to the Society of Critical Care Medicine (SCCM) definition valid since 2010, the term Post-Intensive-Care-Syndrome (PICS) summarizes all new or worsened physical, cognitive or mental impairments that occur after ICU treatment [15]. Fatigue, chronic pain or sleep disorders [16] are also included in this syndrome. The term PICS was not introduced as a medical diagnosis, but rather describes a concept to summarize impairments after the intensive care unit, to foster educational measures and to create awareness [15, 17].

### Empirical findings on the relationship between LC, PSS and PICS

**Fig. 1:**
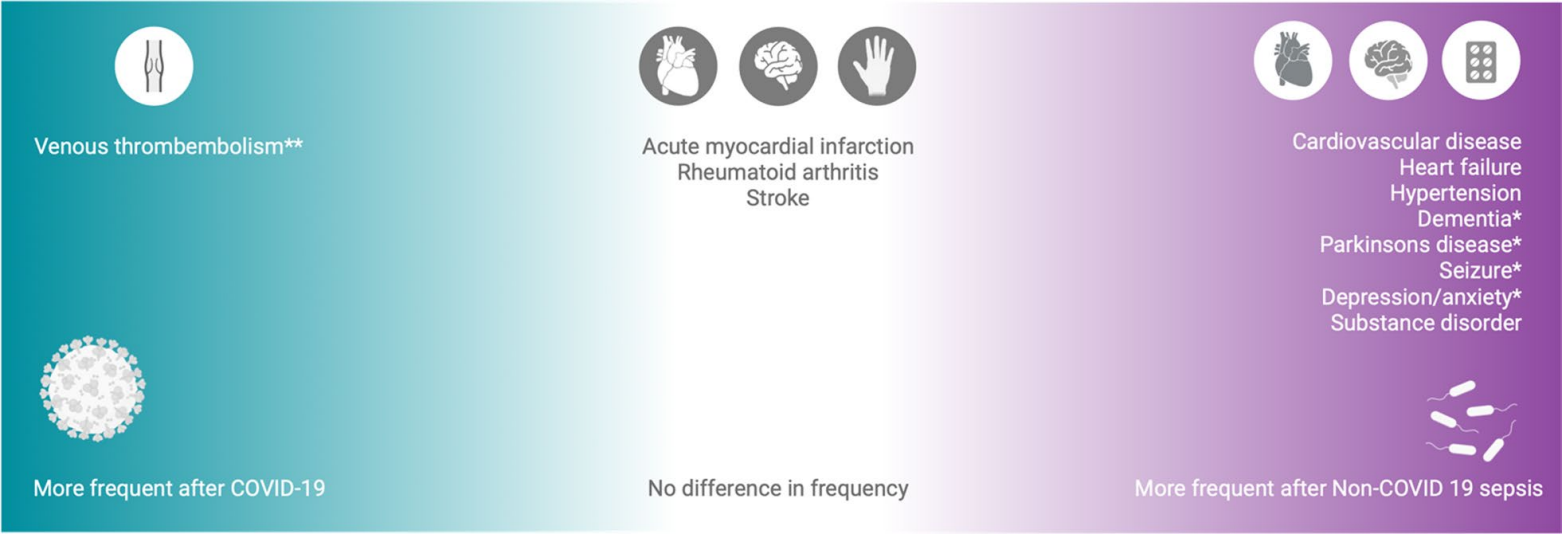
Spectrum of long-term sequelae after COVID-19 compared to Non-COVID-19 sepsis according to Quinn et al. [18]. Legend: *—difference observable only in comparison to historical sepsis cohort, **—difference observable in the 30 days post-discharge. Figure created with BioRender.com

The (co-)occurrence of cognitive, mental and physical disorders characterizes LC, PSS and PICS, with patient-specific symptom spectrums and degrees of severity. With regard to LC and PSS, a cohort study from Canada found that thromboembolic consequences occur more frequently in the 30 days after COVID-19, but cardiovascular consequences (heart failure or hypertension), dementia and depression were more common after non-COVID-19 sepsis in the year after hospital discharge (Fig. 1) [18]. A prospective Danish cohort study reported that cognitive and psychological sequelae did not differ significantly between patients with Covid-19, pneumonia and critical illnesses of other causes [19].

It is not surprising that ICU treatment for Covid-19 increases the likelihood of sequelae, which can also be understood as an expression of the influence of higher disease severity [20, 21]. According to a recent meta-analysis, the odds ratio for the occurrence of Long-Covid was 2.37 (95% confidence interval (CI) 2.18–2.56) in ICU-treated compared to non-ICU-treated patients [20]. The quality of life of survivors was also lower after treatment in the ICU than after Covid-19 disease without ICU treatment [22, 23], and the recovery or regression of symptoms was slower [24]. In addition, the Long-Covid symptom severity after ICU treatment also appears to be higher than in patients without ICU treatment [25].

Furthermore, there are sequelae that arise as a result of therapies carried out during ICU treatment or their complications [26]. One example is the presence of delirium, which can occur as a complication during intensive care treatment due to predisposing (e.g. age, previous illnesses) and precipitating (e.g. severity of illness, medication, mechanical ventilation) factors [26]. Delirium is associated with long-term cognitive impairment [27, 28]. On the other hand, sequelae seem to differ between ICU patients after Covid-19 and after other illnesses to some degree. A meta-analysis of patients with acute respiratory distress syndrome (ARDS) found that pulmonary function in the first year after the illness did not differ in Covid-19-related ARDS from ARDS due to other causes; however, anxiety, depression and post-traumatic stress disorder (PTSD) had a higher pooled prevalence in non-Covid ARDS [29].

The pathophysiology of LC, PSS, and PICS, one the other hand, exhibits significant overlap, but also distinct differences reflecting varying underlying mechanisms (Table 1). All three syndromes involve persistent immune dysfunction, mitochondrial impairment, and systemic inflammation, leading to multi-systemic impacts [10, 13, 30–37]. They have also in common that they overlap with geriatric diseases and comorbidities that may already have existed before the acute illness [38, 39] and are modulated by patient-, treatment- and environment-related factors.

**Table 1:** Comparison of pathomechanisms in LC, PSS and PICS according to current literature

System/mechanism	Long-Covid [10, 34, 35, 40, 41]	Post-Sepsis-Syndrome [13, 30–33, 42–46]	Post-Intensive-Care-Syndrome [15, 47–49]
Immune system	**Dysregulated immune function with chronic inflammatory and immunosuppressive states**	**Dysregulated immune function with a persistent inflammatory and immunosuppressive state**	May be involved depending on the underlying acute illness
Mitochondrial system	**Mitochondrial dysfunction**	**Mitochondrial dysfunction, including impaired mitochondrial membrane potential, ATP production, and increased reactive oxygen species**	
Viral persistence/reactivation	*Viral persistence with active viral presence, affecting antibody production and recovery, reactivation of other viruses including including herpesviruses such as Epstein-Barr virus and Human Herpesvirus 6 among others, viral neuro-invasion*	*Unclear, but viral reactivation has been demonstrated in the context of acute sepsis*	
Microbiome/virome	**Effects on the microbiome or virome**	**Dysbiosis of the gut microbiome in patients with chronic critical illness after sepsis**	
Autoimmunity	**Autoimmune processes**	**Autoimmune processes**	
Vascular system	**Microvascular blood clotting with endothelial dysfunction**	**Microvascular blood clotting with endothelial dysfunction**	
Central- and peripheral nervous system	**Disturbed signal transmission in the brainstem or vagus nerve; inflammation-induced blood–brain barrier permeability, and direct nerve damage suspected, dysautonomia involving autoantibodies and small fiber neuropathy**	**Cerebrovascular damage in the form of ischemia or infarction, neuroinflammation being promoted by the passage of inflammatory mediators across an impaired blood–brain barrier**	
Iatrogenic and treatment-related factors	May contribute to the pathogenesis depending on treatments received		*Mechanical ventilation, immobilization, drug therapies, for example the use of sedatives, opioids, or medication for the neuromuscular blockade, social isolation, anxiety, and disruption of the sleep–wake rhythm during ICU treatment*

Similarities in pathomechanisms are marked in bold, discrepancies in italic

It must also be taken into account that Covid-19 and other infections such as Ebola, the West Nile virus, influenza or Epstein-Barr virus (EBV) can lead to post-infectious sequelae [50, 51], which can have similar but sometimes pathogen-specific patterns of occurrence [52, 53] or depend on the initial focus of the infection [54]. These post-infectious sequelae can also be exacerbated by ICU treatment [14]. The term “infection-associated chronic conditions” (IACC) was therefore introduced in recent publications as an umbrella term to characterize both LC and PSS as well as other post-infectious chronic diseases [50].

### Current challenges in diagnosis

LC, PSS and PICS are syndromes in the sense of symptom complexes, whereby not all symptoms need to co-occur for a diagnosis. This results in a certain heterogeneity in the manifestations of the individual syndromes. At the same time, there is a considerable overlap in possible symptoms, which hinders the differential diagnosis. To this end, it is also unclear if patients can suffer from more than just one of the three syndromes. Such simultaneous diagnosis is currently subject to definitional restrictions, as LC can only be diagnosed if the symptoms cannot be explained by another diagnosis. However, sepsis or ICU treatment in connection with COVID-19 disease are alternative explanations for such symptoms, if the three syndromes are not clearly distinguishable in their clinical appearance and diagnostically.

Moreover, the overlapping disorders and symptoms of LC, PSS or PICS are largely based on the same or at least very similar pathomechanisms (Table 1). Such essentially identical pathogenesis questions the distinction between LC, PSS and PICS as three distinct entities in the sense of actually different syndromes. Otherwise, the terms LC, PSS and PICS would only be three different labels, assigned based on different patient histories, or different point of views and the respective professional background of the treating physician, but would ultimately denote the same symptom complex.

**Fig. 2:**
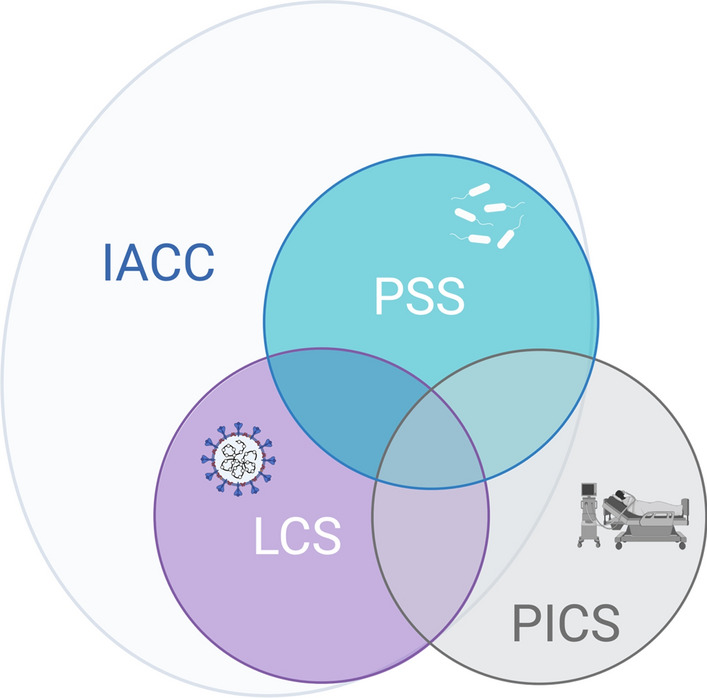
Conceptual illustration of the relation of PICS, PSS and LC symptoms. The Venn diagram shows that there are symptoms that can be attributed to COVID-19, sepsis or the intensive care stay in terms of their etiology. Examples of this are impairments due to viral persistence after COVID-19, or dysphagia after intubation after intensive care stay. There are also symptoms that cannot be specifically assigned, at least not yet, such as depression or cognitive impairment. Figure created with BioRender.com

Currently, we face a lack of diagnostic and clinical differentiability, and unclear distinction do not allow a clear diagnosis of one or more (coincident) syndromes in patients with COVID-19 viral sepsis treated on the ICU due to a lack of diagnostic marker and criteria. We therefore currently assume that many consequences after sepsis cannot be specifically assigned, while only few symptoms are syndrome-specific (Fig. 2) and caused e.g. by COVID-19 persistence in LC.

What are the implications for the clinical treatment of affected patients? The large number of similarities with regard to the underlying pathomechanisms and clinical manifestations suggest or at least increase the probability that therapeutic measures that have proven effective in one of the syndromes will also be helpful in the other two syndromes [55]. More important than the labels PICS, PSS or LC seems to be the diagnosis with regard to the individual complaint and symptom patterns and corresponding targeted therapy offers. Synergies from PSS, PICS and LC research should therefore be increasingly utilized [56, 57].

a) Diagnostics

With regard to PICS, the SCCM recommends early screening of survivors [58]; a core outcome set has been developed for this purpose and short screening questionnaires are also available for clinical use [37, 59, 60]. Various initiatives provide extensive resources, standards and guidelines for the recording of long-term diseases and patient-reported outcome measures (PROMs), such as the "Improve LTO project" [61] or the “Core Outcome Measures in Effectiveness Trials” (COMET) initiative, e.g. providing core outcome measures for physical therapy in the rehabilitation of critical illness survivors after hospital discharge [62].

b) Therapy

The overlap in pathophysiology among LC, PSS and PICS suggests that similar symptom-focused treatment strategies could be beneficial across all three entities. However, developing targeted etiological therapies, like the debated plasmapheresis for LC, depends on understanding disease-specific underlying mechanisms. This approach parallels advances in oncology, where treatments have evolved from broad cytoreductive therapies to precise molecular-targeted interventions. We are at the beginning of such specific therapeutic strategies for LC, PSS, and PICS, and large clinical studies are needed to make significant progress.

In terms of health care structures, the broad spectrum of sequelae after COVID-19, sepsis and ICU treatment results in the need for interdisciplinary, specialized follow-up care that is tailored to the individual needs of patients [63]. According to current guidelines, early mobilization, physical therapy and nutritional or dysphagia management, delirium prophylaxis, ICU diaries and early rehabilitation measures are already recommended in ICU or during the acute hospital stay as part of PICS or PSS prevention or care [64, 65]. In the long term, access to specialized rehabilitation programs should be established [64, 65] and primary care provider should be engaged [66, 67]. The recommendations largely coincide with those for LC [68]. Other aftercare concepts such as app-based rehabilitation services [69], web-based psychotherapy [70], augmented reality-based trauma therapy, community-based follow up (bundles) [71, 72], post-acute treatment bundle strategies [73] or GP-centered case management [74] have been or are being investigated in studies as possible innovations in the area of sequelae after Covid-19, sepsis and ICU treatment; however, with varying degrees of effectiveness or pending results. Furthermore, post-ICU, Long-Covid or Post-Sepsis outpatient clinics, which are usually linked to acute hospitals [75–77], can coordinate post-acute treatment. Particularly for Long-Covid, such structures have been implemented in recent years, with varying operational structures and resources [78]. Here, LC care can benefit from the experience gained in the area of PSS/PICS—and vice versa.

## Conclusions

If long-term impairments occur after ICU treatment for COVID-19 sepsis, these can be caused by the ICU treatment, the septic course with systemic inflammation and organ failure, or by COVID-19 itself. To date, it is not possible to reliably differentiate between LC, PICS and PSS, as the conditions overlap both in terms of etiology, pathogenesis and clinical presentation. Currently, no diagnostic tools are yet available for a reliable differential diagnosis.

**Fig. 3:**
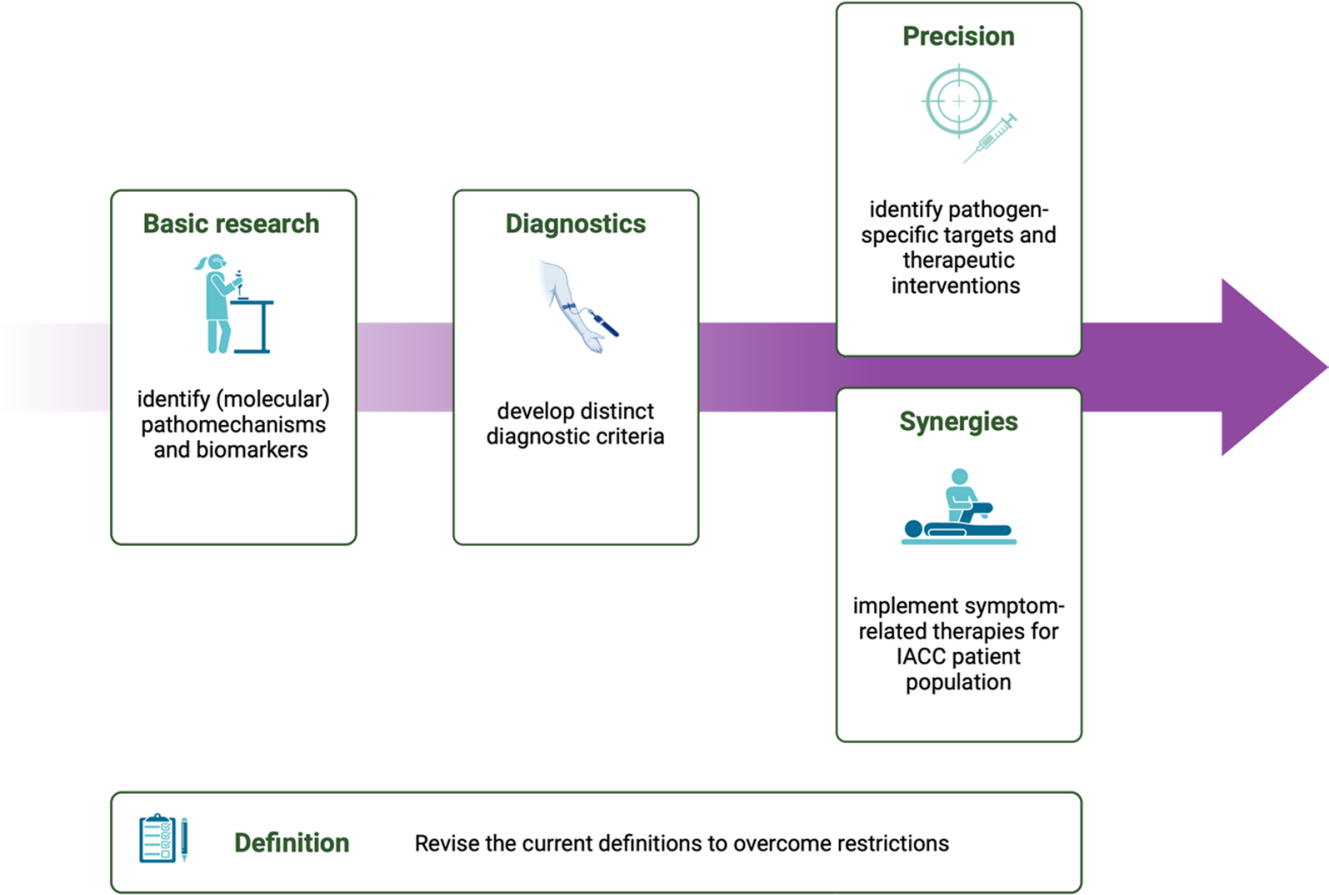
Proposed future research areas on LC, PSS and PICS. Figure created with BioRender.com

If a differentiation of LC, PSS or PICS proves to be clinically meaningful and relevant, future research is needed to understand the exact pathomechanisms and to identify diagnostic criteria that allow both clear diagnoses of each of the three syndromes and the diagnosis of co-incidences between LC, PSS or PICS (Fig. 3). The exclusion of alternative explanations for the respective symptoms of the individual syndromes does not appear to be a suitable criterion.

For clinical treatment, it is relevant that COVID-19 sepsis can have more frequent and more severe consequences than milder COVID-19 courses. COVID-19 sepsis can also lead to different sequelae than sepsis of other origins. For diagnostics and therapy, synergies can arise from PICS, PSS and LC research as well as IACC in general and contribute to optimizing the care of LC, PSS or PICS patients. "Silo" thinking in individual, clearly defined diseases should be overcome, as it may hinder progress and unnecessarily complicate patient care. The focus should be on adequate diagnostics and therapy for all survivors with long-term consequences after COVID-19, sepsis and ICU treatment.

However, advancing clinical management of LC, PSS and PICS does not make the further molecular differentiation of the exact pathomechanisms in LC, PCS and PSS any less important. Exploring these mechanisms is crucial, as it will enable future targeted therapies that go beyond symptomatic treatment in rehabilitation (Fig. 3). For instance, therapies could focus on the elimination of autoantibodies, eradication of viral persistence, and mitochondrial repair, providing more precise and effective treatments tailored to the predominant pathomechanism in each patient. This dual approach of combining broad symptom management with targeted molecular therapies may hold promise for significantly improving patient outcomes.

## Data Availability

No datasets were generated or analysed during the current study.

## Abbreviations

ARDS: Acute respiratory distress syndrome
CI: Confidence interval
COMET: Core Outcome Measures in Effectiveness Trials
COVID-19: Coronavirus disease 2019
EBV: Epstein-Barr virus
GP: General practitioner
IACC: Infection-associated chronic conditions
ICU: Intensive care unit
ICUAW: ICU-acquired weakness
LC: Long-Covid
MDSCs: Myeloid-derived suppressor cells
ME/CFS: Myalgic encephalomyelitis/chronic fatigue syndrome
PASC: Post-acute sequelae after Covid-19
PICS: Post-Intensive-Care-Syndrome
PROMs: Patient-reported outcome measures
PSS: Post-Sepsis-Syndrome
PTSD: Post-traumatic stress disorder
SARS-CoV-2: Severe acute respiratory syndrome coronavirus type 2
SCCM: Society of Critical Care Medicine
